# The raphe nuclear organization and serotonergic system in the bat (*Artibeus planirostris*)

**DOI:** 10.1111/joa.70076

**Published:** 2025-11-16

**Authors:** Mariana D. Leite, Andréa S. M. Bandeira, Wigínio G. L. Bandeira, Milena S. Lustosa, Ana C. F. Gama, Marília A. S. Barros, Paulo L. A. G. Morais, Jeferson S. Cavalcante, Melquisedec A. D. Santana, Expedito S. Nascimento

**Affiliations:** ^1^ Federal Institute of Education Science and Technology of Rio Grande Do Norte Ipanguaçu RN Brazil; ^2^ Department of Morphology/Laboratory of Neuroanatomy, Biosciences Center Federal University of Rio Grande Do Norte Natal RN Brazil; ^3^ BE Bioinsight & Ecoa Porto Alegre RS Brazil; ^4^ State University of Rio Grande Do Norte Mossoró RN Brazil; ^5^ Federal University of Rio Grande Do Norte Natal RN Brazil

**Keywords:** brain stem, chiropteran, comparative neuroanatomy, dorsal raphe nucleus, median raphe nucleus, Phyllostomidae, raphe nucleus, serotonergic system

## Abstract

5‐Hydroxytryptamine (5‐HT), widely recognized as serotonin, is a multifunctional substance present across various body tissues, performing as a neurotransmitter within the nervous framework. Serotonergic neurons are predominantly localized within the raphe nuclei of the brainstem, rendering neuronal 5‐HT a definitive marker for these nuclei. Research has substantiated serotonin's role in the modulation of thermoregulation, appetite, reproductive drive, circadian patterns, slumber, motoric activity, and cognitive processing. While the anatomical structure of serotonergic systems has undergone extensive review in mammalian species, such as rodents, rabbits, felines, and non‐human primates, it remains unexplored in South American bat species. This investigation sought to delineate the serotonergic architecture in the cerebrum of *Artibeus planirostris* through the application of serotonin immunohistochemistry. The study used six adult males *Artibeus planirostris* (family Phyllostomidae, class Mammalia). Following anesthesia and perfusion, their brains were sectioned. Coronal brain sections were acquired using a sliding microtome and processed with an immunohistochemical assay specific to 5‐HT. Caudal linear (CLi), dorsal (DR), median (MnR), paramedian (PMnR), pontine (PnR), magnus (RMg), pallidus (RPa), and obscurus (ROb) raphe nuclei, as well as the B9 and rostral and caudal ventrolateral (rVL/cVL) groups, were marked. Contrary to the serotonergic structures commonly observed in bats, *A. planirostris* dorsal raphe showed a distinct nuclear subdivision, while the median raphe exhibited bilateral representation. The morphometric analysis in this study does not show significant differences in the size of the neuronal area among raphe nuclei. Fresh insights into the field of neuroanatomy are offered by these results, which emphasize potential variations in brain structure among echolocating species in South America as opposed to the more commonly researched models in bats, rodents, and non‐human primates.

## Graphical abstract

Schematic representation of the methodological workflow used to characterize the serotonergic (5‐HT) system in the bat *Artibeus planirostris*. Serotonin (5‐HT) immunohistochemistry was performed on brainstem sections to identify and map the distribution of serotonergic neurons within the raphe nuclei. High‐resolution microscopy and image analysis were used to document the nuclear organization and morphology of labeled neurons.

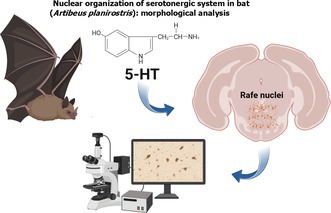

## 1 INTRODUCTION

Bats are the sole mammals capable of flight, displaying great mobility and depending on a diverse range of physical and sensory adaptations (Neuweiler, 2000). These adaptations make it possible for bats to occur in different ecosystems around the world and to explore a wide range of ecological niches (Nowak, 1994). The evolutionary process of adaptive radiation in bats has led to the order Chiroptera being among the most abundant and varied groups of mammals (Altringham, 2011). Due to their high species richness, >1460 species (Simmons & Cirranello, 2020), bats as a group tend to host a great diversity of parasites and, consequently, potentially zoonotic viruses (Mollentze & Streicker, 2020) such as SARS‐like and other coronaviruses (Ge et al., 2016; Li et al., 2005; Zhou et al., 2020). In addition, human‐induced environmental changes, and the decline in biodiversity over recent decades have raised the likelihood of zoonotic transmission and the emergence of diseases (Jones et al., 2013; Keesing et al., 2010). Bats are long‐lived mammals highly tolerant to viral infections (Hayman, 2019; Huang et al., 2019), and the study of anatomical, physiological and molecular mechanisms underlying these traits may have useful applications for human health (Irving et al., 2021; Pereira et al., 2020).

The Phyllostomidae family, characterized by their distinctive leaf‐like nasal structures, presents a compelling case study of adaptive radiation within Mammalia. This highly diverse family, comprising over 200 extant species, originated in the Eocene epoch approximately 50 million years ago within the South American continent. The remarkable diversification observed within Phyllostomidae is primarily attributed to their specialized dietary adaptations. From an insectivorous ancestor, these bats evolved to utilize a wide spectrum of trophic resources, encompassing fruit, nectar, insects, small vertebrates, and even blood (Datzmann et al., 2010).

*Artibeus planirostris*, a member of the Phyllostomidae family, shares a common ancestor with all other neotropical bat leaf‐nosed bats. In contrast to the majority of neotropical chiropteran species, *A. planirostris* exhibits a comparatively larger body mass (40–69 g) (Hollis, 2005). This species demonstrates a wide distribution across both rainforest and seasonal dry forest ecosystems (Barros et al., 2017; Feijó & da Rocha, 2017), functioning as a significant agent of seed dispersal. While the interplay between life history traits and behavioral diversification has demonstrably driven specialized morphological and physiological adaptations in bats, including modifications to nuclear and neuronal organization within conserved brain regions such as the cholinergic, catecholaminergic, and serotonergic systems, as evidenced in pteropodid bats (Dell et al., 2010; Kruger et al., 2010; Maseko et al., 2007; Maseko & Manger, 2007), such investigations remain unexplored within neotropical bats (Phyllostomid).

Recent conceptual advances, summarized under the framework of *serotoninomics* (Jiménez‐García et al., 2025; Jiménez‐Trejo et al., 2023, 2024), emphasize an integrative understanding of the serotonergic system that connects nuclear architecture, gene expression patterns, and behavioral outcomes. This perspective situates the present study within a broader comparative and interdisciplinary context, highlighting how serotonergic nuclei have diversified across mammalian lineages. Within this framework, the analysis of *Artibeus planirostris* offers new insight into the evolutionary and functional adaptations of the serotonergic system in neotropical Phyllostomid bats.

Serotonin (5‐HT) is a crucial neurotransmitter synthesized by particular clusters of cells in the brainstem of researched mammals. It regulates and influences a wide range of functions and behaviors. Studies report that 5‐HT has been linked to mood control (Lowry et al., 2008; Montgomery, 1995), feeding behavior (Takase et al., 2000; Takase & Nogueira, 2008), locomotor function (Noga et al., 2009), regulation of the sleep–wake cycle (Jacobs & Azmitia, 1992; Monti, 2011) sexual behavior, thermoregulation, nociceptive sensory processing, alertness, attention (Jacobs & Azmitia, 1992; Sakai & Crochet, 2001), memory and learning (Vertes & Crane, 1997), circadian rhythm regulation, among others (Jacobs & Azmitia, 1992).

The serotonergic system has been studied morphologically in bats (Dell et al., 2010; Kruger et al., 2010; Maseko et al., 2007; Maseko & Manger, 2007), revealing similarities among studied chiropteran species with slight differences. Curiously, the same pattern has been shown among most mammals across different orders studied to date (Azmitia & Gannon, 1986; Charara & Parent, 1998; Harding et al., 2004; Hornung, 2003; Hornung, 2010; Lidov & Molliver, 1982; Pillay et al., 2017; Soares et al., 2012; Takeuchi et al., 1982; Tork, 1985; Williams et al., 2022), unveiling notable morphological consistency, particularly within eutherian mammals. Thus, while there are differences among species in the quantity of neurons and nuclear divisions, the same core nuclei remain stable. One notable deviation is found in monotremes and fishes (López & González, 2014; Manger et al., 2002), presenting a group of serotonin‐producing neurons in the hypothalamus, a trait not documented in mammals.

The neuronal groups of the raphe, which are the main producers of 5‐HT in the serotonergic system, were initially classified as B1–B9 from the medulla to the mesencephalon in rats (Dahlstrom & Fuxe, 1964). Studies conducted in bats (Dell et al., 2010; Kruger et al., 2010; Maseko et al., 2007; Maseko & Manger, 2007) have revealed all serotonergic nuclei typically assigned to the rostral and caudal clusters identified in rodents and primates. However, one major knowledge gap in bat neuroanatomy is the structure of the serotonergic system in neotropical bats. Most studies on the serotonergic system in bats have been conducted with species found exclusively in the old world (Pteropodid) bat (Kruger et al., 2010; Maseko & Manger, 2007) focusing especially on pteropodidae bats (Dell et al., 2010; Maseko et al., 2007). The present study investigated a neotropical bat species of the family Phyllostomidae, the most ecologically and morphologically diverse group within the order Chiroptera (Altringham, 2011). The nuclear arrangement of the serotonergic system in the brain of *Artibeus planirostris* is detailed here for the first time, a fruit‐eating bat widely distributed throughout South America. Given the significance of the serotonin system for the body and the limited research on the nervous system of neotropical bats, this study seeks to uncover a range of neuroanatomical features of the raphe nuclei. This will contribute to a deeper comprehension of phylogenetic connections between *A. planirostris* and other mammals.

## 2 MATERIALS

### 2.1 Animals and housing

Six adult males *A. planirostris* (body mass range, 40–47 g) captured at the campus of the Federal University of Rio Grande do Norte, Natal, Northeast Brazil, were used in this study, authorized by the Chico Mendes Institute for Biodiversity Conservation (Register No. 25233‐2). All bats were confirmed to be healthy adult males based on body mass, dentition pattern, and gonadal development, and animals showing external lesions or neurological abnormalities were excluded from the study. Bats were captured using three nylon Ecotone® mist nets with dimensions of 3 × 12 m and a mesh size of 19 × 19 mm. The nets were opened after sunset and remained exposed for two consecutive hours. The animals were housed at the Biosciences Center, UFRN, in cages measuring 0.70 × 0.50 × 0.35 m, including 0.15 × 0.13 × 0.29 m nest boxes. The individuals were exposed to controlled light, temperature, and humidity, with food and water freely available. All experimental procedures strictly followed the rules established by the Ethics Committee on Animal Use of the Federal University of Rio Grande do Norte and were approved by this committee (protocol number 009/2012).

### 2.2 Perfusions and section collection

Animals were anesthetized with an intramuscular injection of ketamine (5 mg/kg; Agener), xylazine (0.5 mg/kg; Rhobigarma), diazepam (0.5 mg/kg; Compaz), and tramadol hydrochloride (5 mg/kg; Cristalia) and then perfused transcardially with 150 mL of phosphate‐buffered saline (PBS), pH 7.4, containing 500 UI heparin (Liquemine, Roche, Brazil), followed by 300 mL of 4% paraformaldehyde (Sigma‐Aldrich, USA) in 0.1 M phosphate buffer (PB; Merck, Germany), pH 7.4. After perfusion, brains were removed and immersed in the same fixative solution for 2 h. Finally, for cryoprotection, brains were stored in 30% sucrose solution in 0.1 M phosphate buffer, pH 7.4, for 24–48 h, and then serially sectioned using a freezing microtome, obtaining coronal sections of 30 μm. The combination of ketamine, xylazine, diazepam, and tramadol followed anesthesia protocols validated for chiropteran species to ensure deep analgesia and minimize pre‐perfusion stress (Tavares et al., 2018; Rocha et al., 2020). Tramadol was administered immediately before perfusion to reinforce analgesia and prevent reflex activation during fixation. One brain was sectioned in the sagittal plane. In both cases, the sections (30 μm thickness) were collected sequentially into 6 compartments, each containing one of every 6 sections, thereby representing a serial sequence with a distance of 180 μm between sections in the same compartment.

Sections from one series were immediately mounted on gelatin‐coated glass slides and Nissl‐stained with thionin (García‐Cabezas et al., 2016), to visualize the cytoarchitectonic delimitation of neuronal soma. Sections from another series were submitted to immunohistochemistry to reveal 5‐HT, while the remaining series were utilized for other studies.

### 2.3 Immunohistochemistry

For the detection of 5‐HT, free‐floating sections were incubated for 18–24 h with rabbit anti‐5HT IgG primary antibody (Sigma Laboratories, Inc.), diluted 1:5000, containing 2% bovine serum albumin in 0.4% Triton X‐100 and PBS. Next, the sections were incubated with the biotinylated secondary antibody (goat anti‐rabbit IgG; Jackson Labs, Westgrove, PA, USA), diluted 1:1000, for 90 min, followed by incubation with the avidin‐biotin‐peroxidase solution (ABC Elite kit, Vector Labs, Burlingame, CA, USA) for 90 min.

The reaction was visualized by the addition of diaminobenzidine tetrahydrochloride (Sigma, St. Louis, MO, USA) and 0.01% H_2_O_2_ in PBS. The sections were washed (5x, 5 min) with PBS, between each step and at the end of the procedure. The sections were mounted on glass slides and were then dried, dehydrated in a graded alcohol series, cleared in xylene, and coverslipped with a neutral mounting medium—Erv‐Mount (Erviegas, São Paulo, Brazil).

As a control for reaction specificity, primary antibodies were omitted and replaced with normal goat serum (Vector Laboratories, USA), which completely suppressed immunostaining in all sections.

### 2.4 Digital photography

Digital photomicrographs were captured using a Nikon Eclipse Ni‐U microscope equipped with a DS‐Ril digital camera and processed with NIS‐Elements AR software (Nikon, Japan). Uniform post‐processing was limited to global adjustments of brightness and contrast to preserve image integrity using Canvas 12. Forebrain Atlas of the Short‐tailed Fruit Bat, *Carollia perspicillata* (Scalia et al., 2013) and Cyto‐ and Myeloarchitectural Brain Atlas of the Pale Spear‐Nosed Bat (*Phyllostomus discolor*) in CT Aided Stereotaxic Coordinates by (Radtke‐Schuller et al., 2020) were used to draw nuclear boundaries.

### 2.5 Morphometry

To verify differences in neuronal soma among nuclei, we determined the mean area of the 5‐HT+ soma neurons. The entire rostrocaudal sequence of coronal sections containing serotonergic neurons was examined in each animal and the individual area of each neuron was used to obtain the average of each nucleus. Only neurons with clear soma contours on the same focal plane were considered for analysis. Approximately one hundred neurons were sampled per nucleus. At 20x magnification, we used *ImageJ* software (Java) to make minimal adjustments to brightness and contrast. The Gaussian filter was applied, and the image was binarized to analyze particles.

To provide a comparative morphometric analysis between neurons in the various subdivisions of the studied nuclei, we considered large neurons to be those with areas higher than 70 μm^2^, medium neurons to be those with areas between 60 and 69 μm^2^, and small neurons to be those with areas lower than 60 μm^2^.

The results are given as the mean ± SEM. Normality was determined using the Shapiro–Wilk test in *SPSS 20 IBM* software. Significant differences were assessed using one‐way ANOVA with post hoc analysis by Tukey's test. A corrected *p*‐value of <0.05 was considered statistically significant.

## 3 RESULTS

**FIGURE 1:**
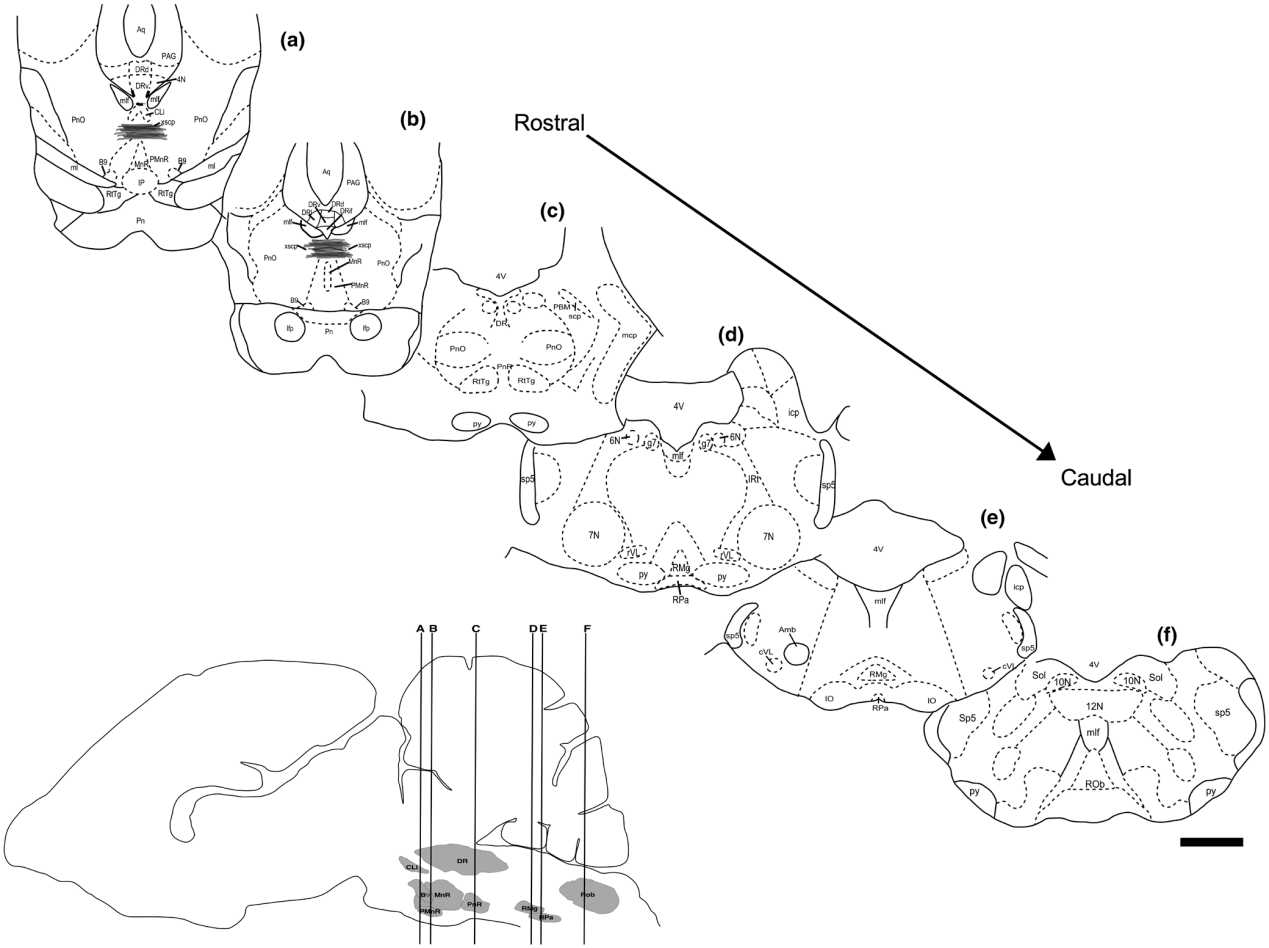
Drawings of coronal sections through the *A. planirostris* brainstem depicting the location of the main 5‐HT‐immunoreactive neuronal groups. At the bottom left is a drawing of a sagittal section through the *A. planirostris* brain, representing the sectional level shown in the coronal sections above. Scale bar: 500 μm. See the list for abbreviations.

This study used 5‐HT immunohistochemistry to map out the serotonergic nuclei in the *A. planirostris* brainstem. Positive for serotonin (5‐HT) neurons are depicted in photomicrographs of immunostained coronal sections obtained from various levels of a typical animal. Localization of 5‐HT‐IR neurons was identified by comparing sections with those in the brain of the Short‐tailed Fruit Bat (*Carollia perspicillata*), as well as the pale spear‐nosed bat (*Phyllostomus discolor*) atlases. 5‐HT‐IR neurons were found throughout the brainstem, mainly in the midline, from the middle portion of the interpeduncular nucleus to the spinomedullary transition. According to the current mammalian brain organization, the 5‐HT neuronal collections are grouped into rostral and caudal clusters. The charts from neighboring coronal Nissl‐stained sections depict the serotonergic clusters in the brainstem of *A. planirostris* (Figure 1).

Statistical analysis of the morphometric data showed no significant differences in neuronal soma areas within the raphe nuclei (*p* > 0.05). Although variations were observed across individual specimens, the overall analysis did not reveal any consistent patterns of significance (Table 1).

**TABLE 1:** Morphometrical analysis.

Animal	B9	RPa	RMg	ROb	DR	CLi	VL	PnR	MnR	PMnR
	Mean ± SD (μm^2^)	Mean ± SD (μm^2^)	Mean ± SD (μm^2^)	Mean ± SD (μm^2^)	Mean ± SD (μm^2^)	Mean ± SD (μm^2^)	Mean ± SD (μm^2^)	Mean ± SD (μm^2^)	Mean ± SD (μm^2^)	Mean ± SD (μm^2^)
M3	57.9 (±25.9)	71.4 (±28.6)	59.7 (±18.2)	55.4 (±16.3)	54.6 (±24.0)	59.9 (±21.7)	70.2 (±30.3)	54.3 (±17.1)	54.3 (±23.5)	57.9 (±19.7)
M5	67.7 (±25.5)	70.8 (±4.5)	67.6 (±29.7)	62.5 (±22.7)	70.9 (±36.6)	72.5 (±36.8)	71.6 (±24.6)	62.4 (±22.1)	68.3 (±26.5)	71.4 (±25.8)
M7	59.1 (±24.9)	64.1 (±25.0)	66.0 (±26.5)	56.9 (±23.8)	63.4 (±40.4)	55.9 (±24.1)	66.5 (±23.1)	46.8 (±19.5)	60.6 (±31.0)	54.3 (±14.1)
M10	60.2 (±24.8)	74.6 (±64.8)	61.1 (±21.7)	50.8 (±17.0)	71.0 (±42.5)	59.1 (±29.5)	78.1 (±25.0)	45.7 (±17.0)	71.5 (±36.2)	59.9 (±19.5)
M13	63.2 (±30.9)	70.7 (±29.1)	65.8 (±24.5)	61.4 (±20.9)	67.2 (±31.9)	59.5 (±21.8)	54.9 (±10.2)	51.1 (±23.1)	66.7 (±31.3)	59.5 (±2.9)
Mean	61.6 (±3.9)	70.3 (±3.8)	64.0 (±3.4)	57.4 (±4.7)	65.4 (±6.8)	61.3 (±6.4)	68.2 (±8.5)	52.0 (±6.7)	64.2 (±5.4)	60.6 (±6.4)

*Note*: Mean values of cellular area and standard deviations in the investigated cases per subdivision.

Although section thickness (30 μm) could theoretically influence soma‐area estimation, all measurements were performed on well‐focused neurons with clearly defined somatic boundaries. Sampling protocols and imaging parameters were applied consistently across all nuclei to minimize underestimation bias, following stereological recommendations for morphometric analysis (Azmitia & Gannon, 1986).

### 3.1 Rostral cluster

**FIGURE 2:**
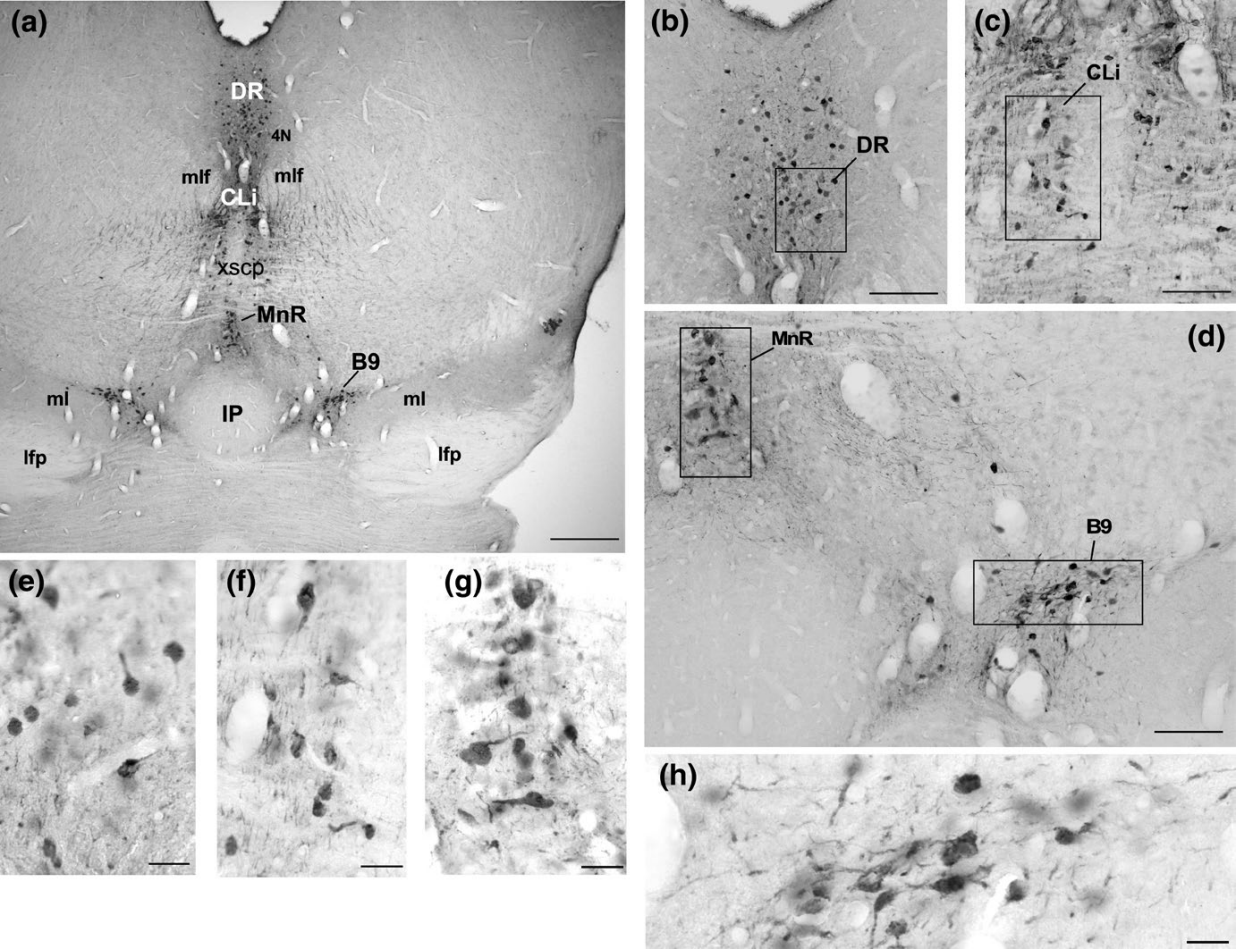
Photomicrographs of 5‐HT‐immunostained brainstem coronal sections illustrating rostral serotonergic cluster, including the dorsal raphe (DR), caudal linear (CLi), the raphe median (MnR), and B9 nuclei in (a). Boxed areas in (b), (c), and (d) are shown in high magnification in (e), (f), (g), and (h) representing characteristic neurons in DR, CLi, MnR, and B9, respectively. See the list for other abbreviations. Scale bar 500 μm (a), 150 μm (b, c, and d), and 60 μm (e, f, g, and h).

**FIGURE 3:**
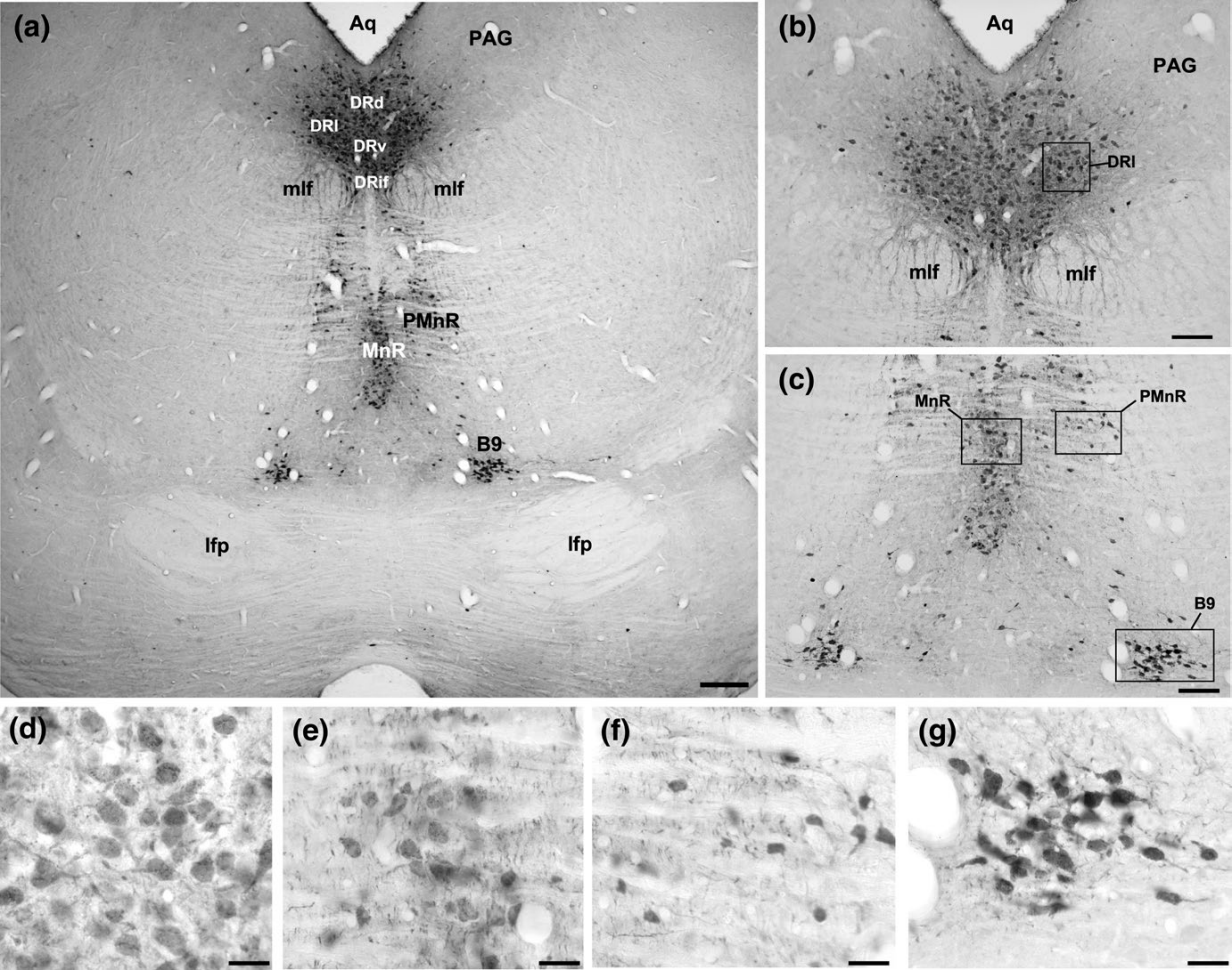
Photomicrographs of 5‐HT‐immunostained brainstem coronal sections illustrating rostral serotonergic cluster, including the dorsal raphe (DR), median raphe (MnR), the paramedian raphe (PMnR), and B9 nuclei in (a). Boxed areas in (b) and (c) are shown in high magnification in (d), (e), (f), and (g), representing characteristic neurons in DR, MnR, PMnR, and B9, respectively. See the list for other abbreviations. Scale bar 200 μm (a), 100 μm (b, c, and d), and 60 μm (e, f, and g).

**FIGURE 4:**
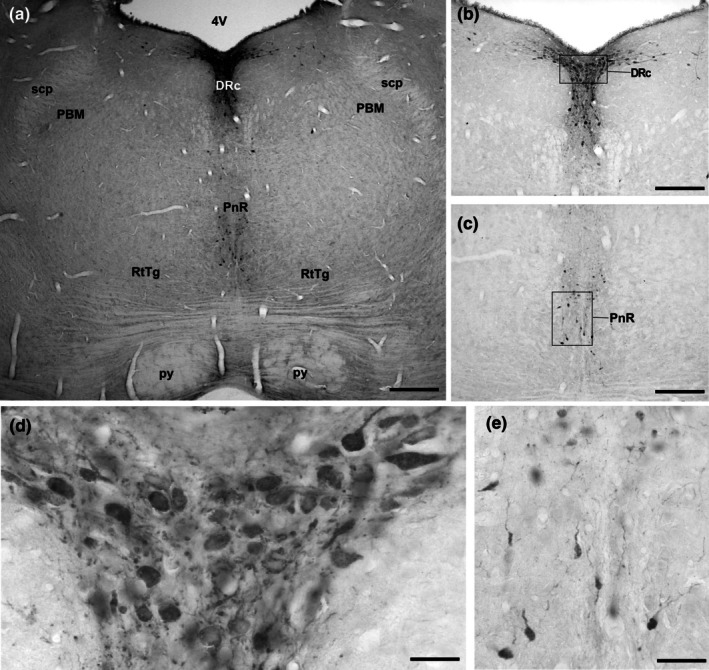
Photomicrographs of 5‐HT‐immunostained brainstem coronal sections illustrating the rostral serotonergic cluster, including the dorsal raphe (DR), pontine raphe (PnR), nuclei in (a). Boxed areas in (b) and (c) are shown in high magnification in (d) and (e), representing characteristic neurons in DR and PnR, respectively. See the list for other abbreviations. Scale bar 200 μm (a), 100 μm (b and c), and 60 μm (d and e).

Serotonergic neurons in the rostral cluster extend from the level of the trochlear nucleus (4 N) periaqueductal gray matter (PAG) in the midbrain to the level of the pyramidal tract (Py) (Figure 1a–c). In *A. planirostris*, the identified nuclei in this cluster include the CLi, B9, MnR, PMnR, DR, and PnR. The RLi was not identified in *A. planirostris* (Figures 2, 3, 4).

#### 3.1.1 Caudal linear nucleus (CLi) and supralemniscal nucleus (B9)

In *A. planirostris*, the CLi was located dorsally to the decussation of the superior cerebellar peduncle (xscp) and ventral to the medial longitudinal fasciculus (mlf) at the midline (Figure 2a). The CLi was triangular‐shaped, with few neurons intermingled with fibers of the xscp. Neurons in the CLi were medium‐sized and ovoid‐shaped (61.3 μm^2^ ± 6.4) compared to the other serotonergic nuclei (Figure 2f and Table 1). Just above the medial lemniscus (ml), a cluster of neurons expands laterally at the same level as the CLi. This cluster was classified as B9 in the present study. (Figures 2h and 3g). Neurons in the B9 were quite like those in the CLi, showing comparable areas and sizes (61.6 μm^2^ ± 3.9) (Table 1).

#### 3.1.2 Median and paramedian raphe (MnR and PMnR)

The median raphe nucleus (MnR) was located at the midline, ventral to the decussation of the superior cerebellar peduncles (xscp), at the same coronal level as the longitudinal fasciculus of the pons (lfp) and dorsal to the interpeduncular nucleus (IP) in rostral sections (Figures 2a and 3a,c). The MnR consists of neurons that form a compact cellular column oriented dorsoventrally at the midline, with clearly defined lateral boundaries. Additionally, some neurons were displaced into lateralized vertical columns known as the paramedian raphe nucleus (PMnR) (Figure 3a,c). The neurons in both the MnR and PMnR were oval‐shaped and medium‐sized, with area measurements averaging 64.2 μm^2^ ± 5.4 for the MnR and 60.6 μm^2^ ± 6.4 for the PMnR (Figures 2g and 3e,f and Table 1).

#### 3.1.3 Dorsal raphe (DR)

The neurons comprising the dorsal raphe complex were mostly associated with the periaqueductal gray matter (PAG), adjacent to the lower border of the cerebral aqueduct (Aq) (Figures 1a,b, 2a, 3a and 4a), extending from the level coinciding with the presence of the trochlear nerve nucleus (4 N) to the parabrachial medial nucleus (PBM) along rostrocaudal extension (Figure 4a). Rostrally, the DR begins as a small cluster of cells surrounded by the PAG and dorsal to the medial longitudinal fasciculus (mlf) (Figure 2a). Caudally, the DR enlarges to form the dorsal subdivision (DRd) immediately ventral to the cerebral aqueduct, with the ventral subdivision (DRv) located further ventrally (Figure 3a). An interfascicular portion (DRif) was observed between the mlf at this level (Figure 3a). Some cells were displaced toward the Aq, lying dorsolaterally to the DRd. Additionally, a packed cluster of 5‐HT‐immunoreactive cells expands laterally from the DRd to form the lateral subdivision (DRl) (Figure 3b,d). The DR terminates at the coronal level where the pontine raphe (PnR) and the reticulotegmental nucleus of the pons (RtTg) emerge. DR cells were oval‐shaped and medium‐sized, averaging 65.4 μm^2^ ± 6.8 (Figures 2e, 3d and 4d, and Table 1).

#### 3.1.4 Pontine raphe (PnR)

At the level of the pons, coinciding with the caudal third of the dorsal raphe nucleus (DR), where the motor trigeminal nucleus and parabrachial complex (PBM) are visible, a cluster of small 5‐HT‐immunoreactive cells, identified as the PnR in *A. planirostris* (52.0 μm^2^ ± 6.7) (Table 1) (Figure 4a,c). PnR neurons were dispersed, ovoid to rounded in shape, and although not statistically different, tended to exhibit smaller somatic profiles than those in the DR. (Figure 4e). This cluster was located between the reticulotegmental nucleus of the pons (RtTg) ventrally and the pontine reticular nucleus (PnO) dorsally.

### 3.2 Caudal cluster

The neurons of the caudal group extend from the junction of the pons and medulla to the junction between the medulla and spinal cord. This grouping includes several nuclear subdivisions, such as RMg, RPa, ventrolateral medullary‐associated neurons (rVL and cVL), and ROb (Figure 1d–f).

#### 3.2.1 Raphe magnus (RMg)

**FIGURE 5:**
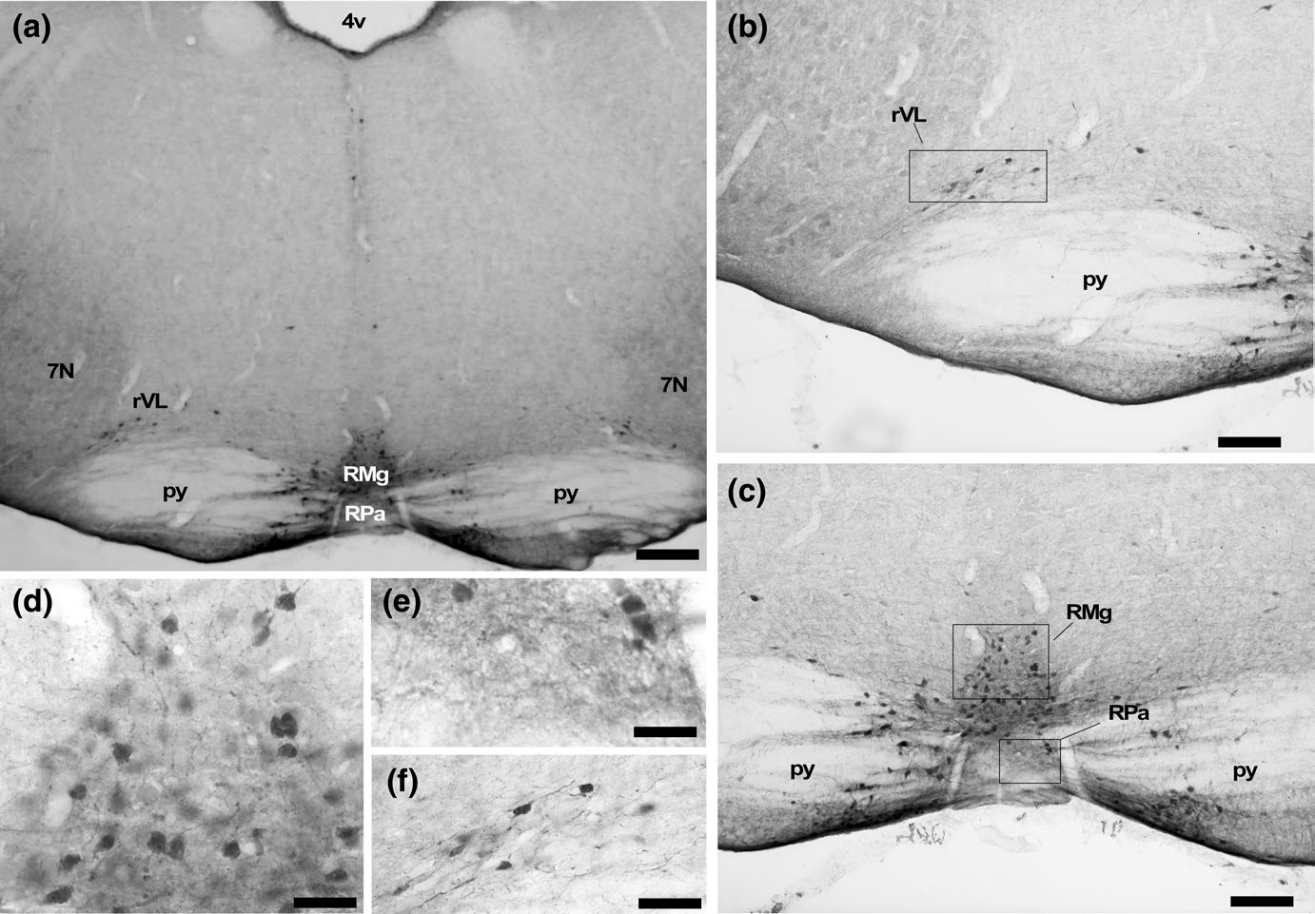
Photomicrographs of 5‐HT‐immunostained brainstem coronal sections illustrating the caudal serotonergic cluster, including the raphe magnus (RMg), raphe pallidus (RPa), and the rostral ventrolateral raphe (rVL) nuclei in (a). Boxed areas in (b) and (c) are shown in high magnification in (d), (e), and (f), representing characteristic neurons in RMg, RPa, and rVL, respectively. See the list for other abbreviations. Scale bar 220 μm (a), 150 μm (b and c), and 80 μm (d, e, and f).

**FIGURE 6:**
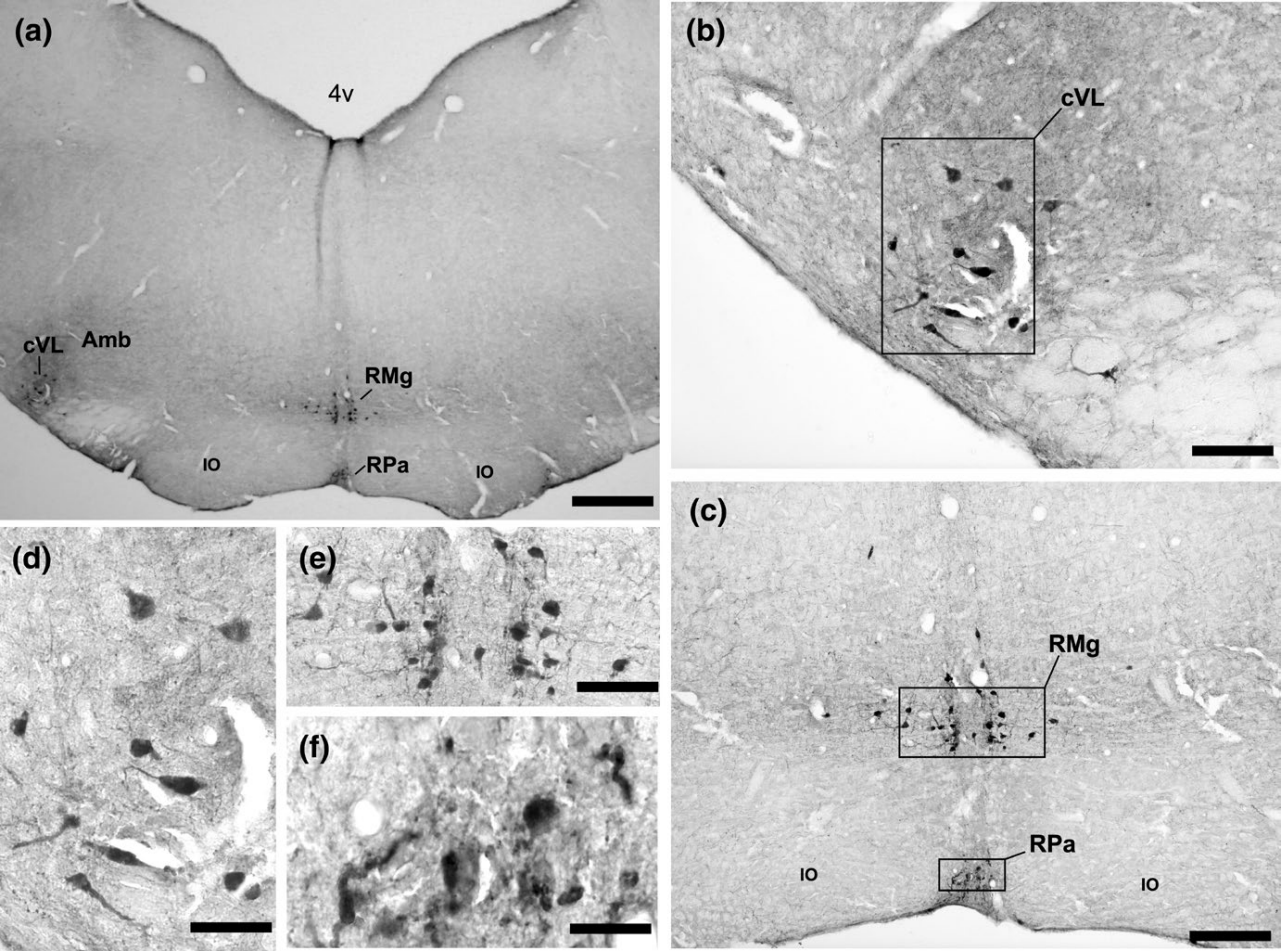
Photomicrographs of 5‐HT‐immunostained brainstem coronal sections illustrating the caudal serotonergic cluster, including the raphe magnus (RMg), raphe pallidus (RPa), and the caudal ventrolateral raphe (cVL) nuclei in (a). Boxed areas in (b) and (c) are shown in high magnification in (d), (e), and (f), representing characteristic neurons in RMg, RPa, and cVL, respectively. See the list for other abbreviations. Scale bar 200 μm (a), 100 μm (b and c), and 70 μm (d, e, and f).

In the rostral part of the medulla, beginning from the rostral level of the spinal trigeminal tract (sp5) and the facial nerve nucleus (7 N), a collection of cells was observed arranged in two columns running parallel to the midline (Figures 5a,c and 6a,c). These neurons constitute the nucleus raphe magnus (RMg) in *A. planirostris* between the two pyramidal tracts (py) in rostral sections. Nevertheless, in caudal sections the RMg was medial to the inferior olive (IO) and dorsal to the raphe pallidus (RPa). The RMg in *A. planirostris* was comprised of medium‐sized neurons, with an average area of 64.0 μm^2^ ± 3.4, displaying rounded and ovoid shapes, as depicted in Figure 5d.

#### 3.2.2 Raphe pallidus (RPa)

Ventral to the raphe magnus nucleus (RMg) and situated between the pyramidal tracts (py), there was a cluster of large serotonergic (5‐HT‐IR) neurons (70.3 μm^2^ ± 3.8) forming the nucleus raphe pallidus (RPa). The inferior olive (IO) delimited the RPa bilaterally at the caudal section. The neurons in the RPa exhibit ellipsoid shapes, as shown in Figures 5a,c,e and 6a,c,f (Table 1).

#### 3.2.3 Rostral ventrolateral (rVL) and caudal ventrolateral (cVL)

At the same coronal levels as the nucleus raphe magnus (RMg) and nucleus raphe pallidus (RPa), there were groupings of laterally scattered 5‐HT‐immunoreactive (5‐HT‐IR) cells known as the rostral ventrolateral (rVL) and caudal ventrolateral (cVL) medullary neurons, respectively (Figures 5 and 6). These populations consist of a small number of cells located away from the midline. The rVL is situated medial to the facial nerve nucleus (7 N) and dorsal to the pyramidal tract (py) (Figure 5a,b), while the cVL is displaced caudally and apparently more lateral to the rVL at the lateral edge of the nucleus ambiguous (Amb) (Figure 6a,b). The neurons in both groups are medium‐sized (68.2 μm^2^ ± 8.5) (Table 1), and exhibit rounded and fusiform shapes (Figures 5f and 6d).

#### 3.2.4 Raphe obscurus (ROb)

**FIGURE 7:**
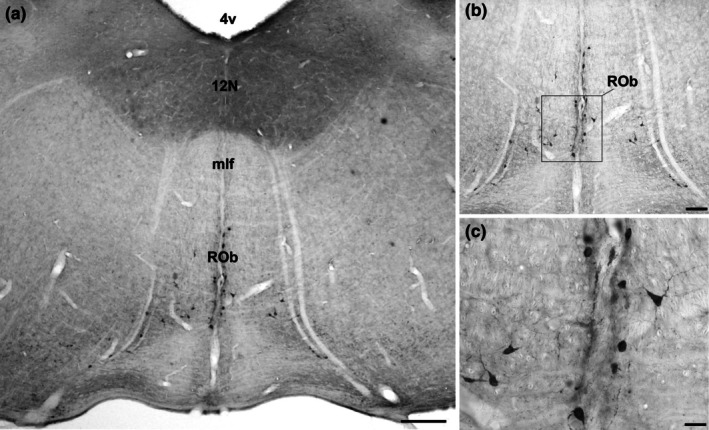
Photomicrographs of 5‐HT‐immunostained brainstem coronal sections illustrating the raphe obscurus nucleus (ROb) in (a). The boxed area in (b) is shown with high magnification in (c), representing characteristic neurons in ROb. See the list for other abbreviations. Scale bar 200 μm (a), 100 μm (b), and 70 μm (c).

The raphe obscurus nucleus (ROb) is the most caudal of the raphe nuclei, located caudally to the nucleus raphe magnus (RMg) at the midline (Figures 1f and 7a). The neurons form two parallel columns of 5‐HT‐immunoreactive (5‐HT‐IR) cells, with some cells displaced laterally (Figure 7b). Topographically, the ROb coincides with the presence of the hypoglossal nucleus (12 N) dorsally. The medial longitudinal fasciculus (mlf) is situated dorsal to the ROb. The ROb extends to the spinomedullary transition. Neurons in the ROb are small and rounded, averaging 57.4 μm^2^ ± 4.7 (Figure 7c and Table 1).

Comparative information across species has been summarized in Table 2, which contrasts the organization of serotonergic nuclei in *Artibeus planirostris* with that reported for rodents, primates, and old world (*Pteropus giganteus*) bats.

**TABLE 2:** Comparative organization of serotonergic nuclei across selected mammalian species.

Order/suborder	Family	Species (Latin)	Common name	Primary reference (year)	Rostral nuclei present (CLi, B9, MnR, DRd, DRv, DRif, DRl, DRp, DRc)	Caudal nuclei present (RMg, RPa, ROb, RVL, CVL)	Notable features/remarks
Chiroptera—Megachiroptera	Pteropodidae	Eidolon helvum	Straw‐colored fruit bat	Dell et al. (2010) (J Chem Neuroanat)	All present (CLi, B9, MnR; DRd/DRv/DRif/DRl/DRp/DRc)	All present (RMg, RPa, ROb, RVL, CVL)	DRl relatively larger than in Epomophorus; standard rostral/caudal clusters described
Chiroptera—Megachiroptera	Pteropodidae	Epomophorus wahlbergi	Wahlberg's epauletted fruit bat	Dell et al. (2010) (J Chem Neuroanat)	All present (CLi, B9, MnR; DRd/DRv/DRif/DRl/DRp/DRc)	All present (RMg, RPa, ROb, RVL, CVL)	DRl smaller than in Eidolon; otherwise canonical organization
Chiroptera—Microchiroptera	Megadermatidae	Cardioderma cor	Heart‐nosed bat	Kruger et al. (2010) (J Chem Neuroanat)	All present (CLi, B9, MnR; DRd/DRv/DRif/DRl/DRp/DRc)	All present (RMg, RPa, ROb, RVL, CVL)	B9 strongly expressed; serotonergic complement homogeneous vs. other microchiropterans
Chiroptera—Microchiroptera	Emballonuridae	Coleura afra	African sheath‐tailed bat	Kruger et al. (2010) (J Chem Neuroanat)	All present (CLi, B9, MnR; DRd/DRv/DRif/DRl/DRp/DRc)	All present (RMg, RPa, ROb, RVL, CVL)	Notably small RMg reported; otherwise typical organization
Chiroptera—Microchiroptera	Rhinolophidae	Triaenops persicus	Persian trident bat	Kruger et al. (2010) (J Chem Neuroanat)	All present (CLi, B9, MnR; DRd/DRv/DRif/DRl/DRp/DRc)	All present (RMg, RPa, ROb, RVL, CVL)	CVL particularly small; rostral cluster fully expressed
Chiroptera—Microchiroptera	Molossidae	Chaerophon pumilus	Little free‐tailed bat	Kruger et al. (2010) (J Chem Neuroanat)	All present (CLi, B9, MnR; DRd/DRv/DRif/DRl/DRp/DRc)	All present (RMg, RPa, ROb, RVL, CVL)	Rostral and caudal clusters present across species; homogeneous appearance
Chiroptera—Microchiroptera	Hipposideridae	Hipposideros commersoni	Commerson's leaf‐nosed bat	Kruger et al. (2010) (J Chem Neuroanat)	All present (CLi, B9, MnR; DRd/DRv/DRif/DRl/DRp/DRc)	All present (RMg, RPa, ROb, RVL, CVL)	Canonical serotonergic complement akin to other microchiropterans
Rodentia	Muridae	Mus minutoides	African pygmy mouse	Kruger et al. (2012) (J Chem Neuroanat)	All present (CLi, B9, MnR; DRd/DRv/DRif/DRl/DRp/DRc)	All present (RMg, RPa, ROb, RVL, CVL)	No reduction in nuclear complement despite very small brain size; RVL/CVL continuous
Primates	Hylobatidae	Hylobates lar	Lar gibbon	Williams et al. (2022) (Anat Rec)	All present (CLi, B9, MnR; DRd/DRv/DRif/DRl/DRp/DRc)	All present (RMg, RPa, ROb, RVL, CVL)	Typical primate‐like organization; DRl limited ventrolaterally vs. chimpanzee
Primates	Hominidae	Pan troglodytes	Chimpanzee	Williams et al. (2022) (Anat Rec)	All present (CLi, B9, MnR; DRd/DRv/DRif/DRl/DRp/DRc)	All present (RMg, RPa, ROb, RVL, CVL)	Expanded DRl (lateral division of DR) noted compared to lar gibbon and other primates
Chiroptera—Microchiroptera	Phyllostomidae	*Artibeus planirostris*	Flat‐faced fruit‐eating bat	This study (Journal of Anatomy)	CLi, B9, MnR, DRd, DRv Drif, DRl, DRp, DRc	RMg, RPa, ROb, RVL, CVL	Enlarged Rpa with large neuron and compact DRv subdivision. Partial condensation of the caudal raphe complex and subtle reorganization within the DR, reflection ecological adaptation of neotropical Phyllostomids

*Note*: Summary of the presence and relative differentiation of the classical serotonergic nuclei (rostral: CLi, B9, MnR, and DR subdivisions; caudal: RMg, RPa, ROb, RVL, CVL) in representative Megachiropteran, Microchiropteran, Rodent, and Primate species. The table highlights interspecific variations in nuclear size, distribution, and morphology, as reported in previous neuroanatomical studies. Data for *Artibeus planirostris* (this study) show a relatively enlarged RPa and compact DRv subdivision compared with other bats, suggesting subtle reorganizations of the caudal and dorsal raphe complexes in neotropical Phyllostomids.

## 4 DISCUSSION

This study provides the first comprehensive overview of the distribution of serotonin‐producing neuronal cell clusters in the brainstem of a notable South American bat, *A. planirostris*, using 5‐HT immunohistochemical staining. Direct comparison with Nissl‐stained material in alternating sections allowed us to associate 5‐HT‐IR cells with their respective cytoarchitectonic territories related to the raphe nuclei in the *A. planirostris* brainstem. Further, we used the Forebrain Atlas of the Short‐tailed Fruit Bat, *Carollia perspicillata* (Scalia et al., 2013) and the Cyto‐ and Myeloarchitectural Brain Atlas of the Pale Spear‐Nosed Bat (*Phyllostomus discolor*) in CT Aided Stereotaxic Coordinates (Radtke‐Schuller et al., 2020) to identify raphe nuclei in the *A. planirostris* brainstem. These include the caudal linear raphe, dorsal raphe, median and paramedian raphe, pontine raphe, raphe pallidus, as well as 5‐HT‐IR neurons observed in lateral clusters such as B9 and the ventrolateral medulla oblongata region.

To our knowledge, this is the first detailed description of the serotonergic system in a neotropical bat species. Previous studies on serotonergic neuronal distribution in Chiroptera have been restricted to old world taxa, including *Rousettus aegyptiacus*, *Eidolon helvum*, *Epomophorus wahlbergi*, *Miniopterus schreibersii*, and other African or paleotropical species (Maseko & Manger, 2007; Maseko et al., 2007; Maseko & Manger, 2007). The present findings in *Artibeus planirostris* thus expand the neuroanatomical knowledge of the serotonergic system to the Neotropical radiation of bats, providing a valuable comparative framework for understanding the evolutionary diversification of monoaminergic networks within Chiroptera.

Recent evidence emphasizes that serotonergic neurons exhibit substantial molecular and functional heterogeneity, comprising several transcriptionally distinct subpopulations with specific projection targets (Huang et al., 2019; Okaty et al., 2015). This diversity could underlie species‐specific patterns of serotonergic modulation and may contribute to the adaptive neural traits observed in Neotropical bats. In this regard, genomic data on chiropteran evolution (Nikaido et al., 2020) provide complementary insights that can help correlate serotonergic organization with evolutionary divergence within the order Chiroptera.

The distribution of serotonergic nuclei has been investigated through immunohistochemistry procedures in numerous species. Two species of marsupials, the opossum, and the wallaby, have serotonergic positive neurons in two clusters in their midline (Crutcher & Humbertson, 1978; Ferguson et al., 1999). The rostral cluster includes CLi, MnR, PnR, and DR, while the caudal one consists of RMg, RPa, and ROb. Additionally, there are laterally positioned cells known as the B9 subdivision. Serotonergic cell distribution and morphology were also studied in two monotreme species: the platypus and the short‐beaked echidna (Manger et al., 2002). Curiously, monotremes display a group of serotonin‐producing cells in the hypothalamus, which was previously identified only in non‐mammalian vertebrates like fish, birds, and reptiles (Ikeda & Goto, 1971; Parent & Northcutt, 1982; Wolters et al., 1985). Furthermore, a broad number of studies have been conducted in rodents (Dahlstrom & Fuxe, 1964; Fuxe et al., 1969; Harding et al., 2004; Hornung, 2010; Oades & Halliday, 1987; Soares et al., 2012; Steinbusch, 1981), as well as in primates (Azmitia & Gannon, 1986; Hornung, 2003; Hornung & Fritschy, 1988; Williams et al., 2022).

The organization of serotonergic nuclei was documented in five Microchiroptera species. These studies revealed a consistent arrangement of serotonergic nuclei within the rostral and caudal clusters, aligning with observations in most eutherian mammals. Notable exceptions include the sparse distribution of 5‐HT positive neurons within the caudal magnus raphe nucleus of *Coleura afra* and a reduced neuronal population within the caudal ventrolateral group of *Triaenops persicus* (Kruger et al., 2010). In the remaining examined species, these nuclei exhibited comparable characteristics to those reported in other mammalian studies. Broadly, the serotonergic raphe nuclei of *A. planirostris* resemble those observed in other investigated chiropterans, albeit with subtle distinctions. Our study identified a substantial presence of large neurons within the pallidus raphe nucleus and small neurons within the obscurus raphe nucleus, contrasting with observations in *Coleura afra* and *Triaenops persicus*. Additionally, the MnR in *A. planirostris* presents as a distinct cellular column, mirroring the morphology described in *Phyllostomus discolor* (Radtke‐Schuller et al., 2020) and diverging from the organization observed in *Coleura afra* and *Triaenops persicus* (Kruger et al., 2010). These interspecies variations may reflect divergent phylogenetic trajectories, environmental pressures, and life history strategies.

Previous investigations into the dorsal raphe nucleus of various bat species have revealed notable interspecies variations. Maseko and Manger (2007) identified six subdivisions within the DR of *Miniopterus schreibersii*, while Kruger et al. (2010) documented a similar six‐part organization in *Chaerophon pumilus*, *Hipposideros commersoni*, *Coleura afra*, and *Triaenops persicus* (Kruger et al., 2010). A comparative analysis of the DR in *Miniopterus schreibersii* and *Hipposideros commersoni* highlights a complementary distribution of neurons. While *Hipposideros commersoni* lacks neurons in the central DR, *Miniopterus schreibersii* exhibits a vacant lateral DR (Kruger et al., 2010; Maseko & Manger, 2007). Furthermore, a laterally positioned cell cluster, previously designated as the lateral DR (lDR), was observed in both species. This group, situated adjacent to the cerebral aqueduct, was characterized as a displaced neuronal population dorsal to the primary DR. A more accurate anatomical description might employ the terms “dorsolateral subdivision” or “periaqueductal subdivision” to delineate this group. Interestingly, our examination of *Artibeus planirostris* revealed four subdivisions with a homogenous distribution of neurons within the DR, lacking the distinct periaqueductal portion observed in previous studies.

Our findings in *Artibeus planirostris* suggest a confluence of the distinct DR organizations previously observed in *Miniopterus schreibersii* and *Hipposideros commersoni*. In contrast to the compartmentalized DR structure reported in other bat species, *A. planirostris* exhibits a compact cluster of 5HT‐immunoreactive neurons that occupy the entirety of the DR subdivisions along its rostrocaudal axis. This observation diverges from prior descriptions of the bat DR. Our analysis does not support the presence of a laterally positioned lDR surrounding the cerebral aqueduct as proposed by Maseko and Manger (2007), and Kruger et al. (2010). Similarly, we found no evidence of the peripheral dorsal raphe subdivision (DRp) described in those studies. Instead, our cytoarchitectonic and immunohistochemical analyses consistently revealed four distinct areas within the *A. planirostris* DR (DRd, DRv, DRl, and DRif). We did not observe a DRl positioned dorsolaterally to the cerebral aqueduct, nor could we confirm the existence of a DRp in this species.

The morphometric analysis conducted in this research found that, although neurons in the RPa of *A. planirostris* appeared larger compared to those in other serotonergic nuclei, there were no significant differences observed among the nuclei analyzed. Curiously, neurons within the PnR also exhibit smaller size in comparison to all serotonergic nuclei found in *A. planirostris*. The absence of significant differences in neuronal nucleus areas within the raphe nuclei suggests a consistent level of morphometric homogeneity in this brain region. Importantly, morphometrical analysis conducted here was the first performed in bats so far. The discoveries add new perspectives to our comprehension of the structural features of the raphe nuclei in bats and emphasize the necessity for similar studies in pteropodidae bats and Neotropical bat species of the family Phyllostomidae in order to compare among species.

Beyond the structural description, the serotonergic organization of *A. planirostris* may have functional implications related to its frugivorous diet, nocturnal habits, and complex social behavior. These ecological variables can shape sensory integration and autonomic regulation mediated by the raphe system, potentially driving subtle morphological specializations in serotonergic nuclei.

From an evolutionary standpoint, it is argued that shifts in neural system complexity, as measured by identifiable subdivisions, occur solely during the emergence of a new mammalian order (Manger, 2005). Once a mammalian order is established, all descendants retain the same additional brain systems as the progenitor of that order, regardless of any subsequent changes in brain size, phenotype, or life history. Consistent with this viewpoint, the serotonergic system shows many similarities across various studied bat species, extending from laboratory rats (Harding et al., 2004) to wild species such as the highveld gerbil (Moon et al., 2007) or the rock cavy (Soares et al., 2012).

Despite minor discrepancies potentially attributable to variations in nomenclature, our findings reveal subtle differences in the organization of serotonergic neurons within the *A*. *Artibeus planirostris* brainstem compared to data from pteropodid bats. These differences are particularly evident in the structure of the dorsal raphe nucleus and the median raphe nucleus. This distinct organization may be linked to divergent life histories between South American Phyllostomidae and old world (Pteropodid) bats, separated by approximately 50 million years of evolution. This divergence underscores the concept of the serotonergic system as a fundamental neurotransmitter network, conserved throughout evolution across diverse species, albeit with subtle adaptations.

## 5 CONCLUSION

This study provides the first detailed neuroanatomical mapping of serotonergic nuclei in a Neotropical bat. While the general organization parallels that of other mammals, *Artibeus planirostris* shows distinctive patterns within the dorsal and median raphe complexes that may represent evolutionary specializations associated with ecological and behavioral traits. Future studies combining serotoninomics and genomic approaches are required to elucidate the functional significance of these neuroanatomical adaptations.

## AUTHOR CONTRIBUTIONS

ESNJ and MDL participated in the conception and planning of this study. MADS had the primary responsibility for all aspects of bat husbandry. MASB and MDL have the responsibility for all aspects of bat capturing and handling. ESNJ and MDL supervised the tissue processing. ASMB, MSL, ACFG, and WGLB supervised the photography, prepared the early drafts of the article and figures, and analyzed the results. ESNJ, MDL, MADS, WGLB, PLAGM, JSC, and MASB have read and made substantive additions and corrections to the final article.

## CONFLICT OF INTEREST STATEMENT

None of the authors has, or has had, any association with any person or organization that could constitute a conflict of interest concerning the conduct or outcome of this work.

## Data Availability

Data from this study are available from the corresponding author upon reasonable request.
